# From In Silico Hypothesis to Validation: The Role of Real-World Evidence in the Preliminary Verification of AI-Generated Drug-Repositioning Candidates: A Comprehensive Review

**DOI:** 10.3390/jcm15072801

**Published:** 2026-04-07

**Authors:** Michał Gałuszewski, Jan Olszewski, Karolina Jankowska, Krzysztof Wójcik, Anna Bielecka-Wajdman

**Affiliations:** 1 Student Scientific Club, Department of Pharmacology Medical, University of Silesia, Medyków 18 St., 40-752 Katowice, Poland; s86715@365.sum.edu.pl (M.G.); s88137@365.sum.edu.pl (K.J.); s87699@365.sum.edu.pl (K.W.); 2 Department of Pharmacology, Medical University of Silesia, Medyków 18 St., 40-752 Katowice, Poland; abielecka-wajdman@sum.edu.pl

**Keywords:** drug repositioning, artificial intelligence, real-world evidence, electronic health records, precision medicine

## Abstract

**Background/Objectives:** Drug repositioning has emerged as a promising strategy to address the innovation crisis in pharmaceutical development. While artificial intelligence enables efficient in silico hypothesis generation, clinical translation remains challenging. This study aims to evaluate the role of Real-World Evidence (RWE) in validating AI-generated drug-repositioning candidates. **Methods:** A comprehensive literature review was conducted in PubMed using a predefined search strategy integrating drug repositioning, artificial intelligence, and real-world data. After multi-stage screening, 22 original research articles were included for analysis. **Results:** Network-based algorithms and natural language processing dominated AI-driven hypothesis generation. Validation using Electronic Health Records and insurance databases enabled retrospective assessment of drug efficacy across large populations. Successful applications were identified in neurodegenerative, metabolic, infectious, autoimmune, and psychiatric diseases. **Conclusions:** The integration of AI-based analytics with RWE provides a promising framework for the preliminary verification of computational predictions, potentially informing the translational pathway toward clinical practice. However, the effectiveness of this approach remains dependent on data quality and the specific therapeutic context, requiring further standardization of clinical data.

## 1. Introduction

The contemporary pharmaceutical sector has been struggling for decades with the so-called innovation crisis, characterized by a decline in productivity despite increasing investment in research. The traditional process of bringing a new drug to market (de novo) is an extremely time-consuming and costly undertaking, lasting on average from 10 to 15 years, with investments ranging from 500 million to even 2 billion dollars [1]. Despite such vast resources, the failure rate remains critically high; it is estimated that approximately 90% of drug candidates are rejected during the clinical trial stage [2]. The primary causes of these failures include safety issues, insufficient exposure at the site of action, and a lack of expected efficacy in humans. Furthermore, unexpected side effects, whose mechanisms often remain unknown, lead to the market withdrawal of even already approved preparations, generating financial losses and posing a threat to patients [1]. In light of these challenges, there is an urgent need to optimize the therapeutic process by reducing costs and risks.

The answer to the limitations of the de novo model is the drug-repositioning strategy (also known as drug repurposing), defined as the process of identifying new therapeutic applications for substances that have already been approved or whose safety has been confirmed [3]. A key advantage of this approach is the fact that the pharmacokinetic (PK) and pharmacodynamic (PD) parameters, as well as the safety profile of the reprofiled candidates, are already known. This allows for the bypassing of early safety research phases, which may significantly reduce development time, lower costs, and potentially improve the probability of success in clinical phases [2]. Medical history knows spectacular examples of this phenomenon, such as sildenafil, originally studied for angina pectoris but utilized in the treatment of erectile dysfunction, or finasteride, redirected from the treatment of prostate enlargement to hair loss therapy [3]. Currently, however, there is a shift away from accidental discoveries (serendipity) toward systematic, rational methods of searching for new indications, which has become possible due to access to large-scale biomedical data.

The transition toward systematic drug discovery has been enabled by the unprecedented, exponential growth in the availability of biomedical data and the development of computational technologies [4]. In recent decades, we have observed a rapid increase in “omics” and health data, which can be collected and analyzed in real-time. The use of artificial intelligence (AI) in the life sciences allows for a move away from traditional research hypotheses in favor of data-driven hypotheses, revolutionizing the process of identifying new therapeutic applications [4]. Computational methods (in silico) enable the rapid processing of these datasets; however, previous approaches focused mainly on the molecular level, which often proved insufficient to map the full complexity of human physiology and the final effects of the drug in the body [5].

To overcome the limitations of purely molecular models, it becomes crucial to incorporate evidence from actual clinical practice (RWE—Real-World Evidence). Electronic Health Records (EHRs) represent a rich source of phenotypic data, enabling the verification of drug action in a diverse patient population rather than only under controlled experimental conditions [6]. Modern approaches utilize these data to search for so-called complementarity (anti-correlation) between the clinical signature of a disease and the drug’s effect profile [5]. The integration of information on real physiological outcomes contained in EHRs with advanced analytics allows for more precise prediction of indications even for “first-in-class” drugs for which previous literature data are lacking, serving as a link between in silico hypotheses and preliminary clinical observation [6].

Ultimately, the greatest potential in modern pharmacology lies not in the isolated use of individual tools but in the synergy of computational methods with clinical data analytics. As Denny et al. point out, the rapid development of large research cohorts linked to EHR data creates the foundation for precision medicine, enabling the tailoring of therapies to individual genetic and phenotypic patient profiles [7]. The aim of this work is to critically examine current evidence for how the integration of large-scale network analyses and ML with RWE may support the efficiency of the repositioning process. This approach allows not only for more precise prediction of the efficacy of new indications [2] but also for the early identification of potential adverse effects, which may contribute to enhanced patient safety [1]. To ensure methodological clarity, the integration of AI and RWE should be viewed as a three-stage pipeline. First, hypothesis generation utilizes computational in silico methods, predominantly network-based algorithms and natural language processing, to identify potential drug-disease associations. Second, retrospective validation leverages large-scale RWE from EHRs and insurance databases to assess drug efficacy and safety profiles across diverse populations. Finally, the goal of this preliminary verification is to provide data-driven insights that may inform the transition from computational theory toward prospective clinical evaluation [2,3]. This review systematizes the evidence regarding how the preliminary verification of in silico hypotheses in an EHR environment may provide population-level signals that assist in prioritizing candidates for further prospective validation.

## 2. Materials and Methods

A targeted literature search was conducted in the PubMed database to identify high-quality clinical studies integrating AI and RWE. PubMed was selected as the primary source due to its comprehensive indexing of peer-reviewed biomedical literature and MEDLINE-indexed journals, which prioritize evidence validated in clinical settings. While this single-database approach ensures a focus on rigorous, peer-reviewed medical data, we recognize that the exclusion of other databases (e.g., Scopus or Web of Science) represents a limitation in the breadth of technical AI models identified. To further assess the comprehensiveness of our strategy, a comparative search was performed in Web of Science (52 records) and Scopus (87 records) using the same keyword combinations. After deduplication and manual screening of titles and abstracts, it was determined that the additional records from these databases consisted primarily of technical conference proceedings or purely computational studies that did not meet our strict inclusion criteria for primary clinical RWE validation. Thus, the restriction to PubMed ensured a focused synthesis of high-quality, peer-reviewed clinical evidence indexed in MEDLINE. The query was completed on 30 October 2025. The search strategy was based on a combination of keywords and Medical Subject Headings (MeSH) terms covering three main subject areas: drug repurposing, computational methods, and real-world data.

**Figure 1:**
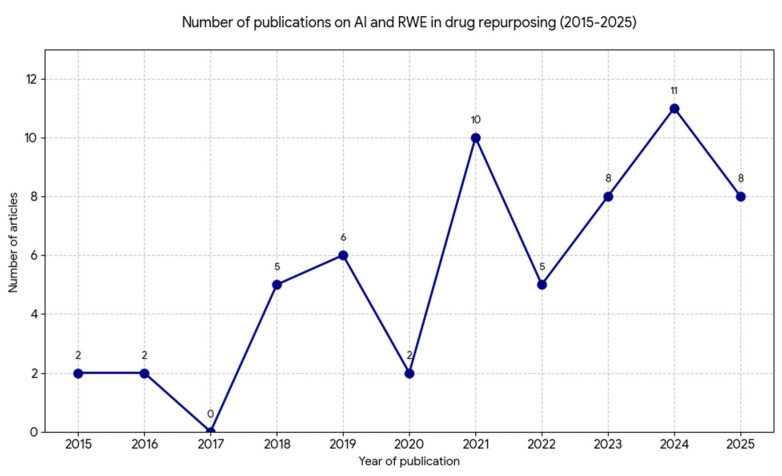
Number of articles published between 2015–2025 in PubMed (as of 30 October 2025) (own study).

The following search string was used: (“drug repurpos”[tiab] OR “Drug Repositioning”[Mesh]) AND (“artificial intelligence”[tiab] OR “machine learning”[tiab] OR ‘computational’[tiab]) AND (“Real-World Evidence” [tiab] OR “Real-World Data”[tiab] OR “electronic health records”[Mesh] OR registry[tiab] OR “claims data”[tiab] OR observational[tiab] OR “cohort”[tiab]). The number of publications broken down by year is shown in Figure 1.

**Figure 2:**
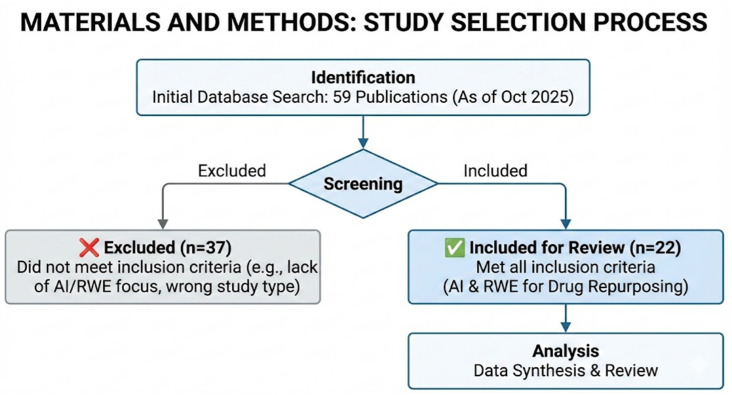
Search and selection flowchart illustrating the targeted identification and inclusion of studies for this review (*n* = 22).

A preliminary search of the database identified 59 records. All articles underwent a multi-stage selection process. In the first phase, titles and abstracts were verified for relevance to the topic of the study, resulting in the rejection of non-relevant items, duplicates, and works without full text available in English. After a detailed full-text analysis, 38 papers that did not meet the inclusion criteria (e.g., lack of an RWE component or lack of AI-based methodology) were excluded from further review. Ultimately, 22 articles were selected for qualitative analysis. A detailed diagram of the selection process is presented in Figure 2.

During the preparation of this manuscript, the authors used AI tools (Gemini 3 Flash by Google) for identifying relevant research data and for linguistic correction and stylistic refinement. Following the peer-review process, the manuscript underwent an additional round of comprehensive linguistic polishing and structural verification to ensure the highest clarity of the scientific content. The authors have reviewed and edited the final output and take full responsibility for the content of this publication.

The literature selection was based on strictly defined inclusion criteria. Only original research papers published in English and available in full text were included in the review. The key substantive criterion was the presence of an integrative component: articles that presented the use of artificial intelligence (AI) or machine learning (ML) for drug repositioning, while using real-world data (RWE/EHR) as a source of input data or a method for validating in silico hypotheses. Publications that did not meet these conditions, including purely theoretical works, review articles, and studies without an RWE/AI component, were excluded from further analysis.

## 3. Results

Strategies for finding new indications for existing drugs can be systematically divided into three main research streams: drug-centric, target-centric, and disease-centric [2]. Although historically many groundbreaking discoveries have resulted from accidental clinical observations or in vitro experiments, modern pharmacology is evolving towards rational, data-driven design. This change is driven by the exponential growth of biomedical information—from genomic data to scientific literature—whose effective analysis exceeds human cognitive capabilities [4]. As a result, computational methods have become an indispensable part of this process. Among them, network-based approaches, text mining, and machine learning (ML) algorithms play a dominant role, enabling the detection of hidden patterns and the generation of hypotheses with a high probability of clinical success [3].

### 3.1. Alzheimer’s Disease

Although β-amyloid deposits and neurofibrillary tangles represent the histopathological hallmark of Alzheimer’s disease (AD), the pathobiological understanding of this disorder has expanded significantly due to new molecular discoveries [8]. In 2016, the number of people living with dementia hovered around 48.3 million, compared to 20.2 million in 1990, representing more than a two-fold increase [9]. Despite intensive research, progress in pharmacotherapy remains unsatisfactory. The latest meta-analysis from 2025 (Jeremic et al.) indicates that even innovative monoclonal antibody therapies (such as donanemab or lecanemab), while reducing amyloid levels, offer only moderate slowing of clinical disease progression and carry the risk of serious adverse effects [10]. To identify optimal therapeutic options, AI based on large datasets can assist by analyzing new drugs that may serve in treating Alzheimer’s disease [11,12,13,14,15].

In a study conducted by Taubes et al. (2021), a precision medicine approach was applied, focusing on the APOE4 genotype, which is the most significant genetic risk factor for late-onset AD. Using transcriptomic analysis, bumetanide was identified as a leading repositioning candidate capable of reversing the pathological gene expression characteristic of APOE4 carriers. Transcriptomic analysis isolated a specific signature of 539 genes in patients with the APOE4/4 genotype, for which computational algorithms identified bumetanide as the candidate with the highest therapeutic potential. Verification in the J20/E4-KI mouse model confirmed that bumetanide treatment leads to a statistically significant reduction in the number of amyloid plaques in the hippocampus (*p* = 0.0478) and cerebral cortex (*p* = 0.0442), as well as a reduction in their surface area (*p* = 0.0357 and *p* = 0.0432, respectively). The therapy also restored normal synaptic plasticity, as demonstrated by an increase in LTP (*p* < 0.0001) and improved cognitive function in behavioral tests (*p* = 0.0345). The drug’s translational potential was confirmed by an analysis of medical data from over 5 million patients, showing a 35–75% reduction in the incidence of Alzheimer’s disease among individuals over 65 years old using bumetanide, which was statistically significant in two independent cohorts (*p* = 0.0333 and *p* = 1 × 10^−8^) [11].

In a study performed by Fang et al., a novel framework based on artificial intelligence and network medicine was employed, using a Bayesian algorithm to integrate GWAS results with multi-omic data, which allowed for the identification of 103 AD risk genes (ARGs) with high therapeutic potential. Population validation showed that the use of pioglitazone is associated with a significant reduction in AD risk compared to a matched control group (HR = 0.916; 95% CI 0.861–0.974; *p* = 0.005). This effect was also confirmed in comparison with glipizide, another antidiabetic drug (HR = 0.921; 95% CI 0.862–0.984; *p* = 0.0159), suggesting a specific neuroprotective action. In vitro mechanistic studies on human microglial cells demonstrated that pioglitazone reduces the activity of GSK3β and CDK5 kinases, indicating a potential mechanism for its beneficial effect. In addition to pioglitazone, a significant association with reduced AD risk was also shown for febuxostat and atenolol [12].

Xiang et al. (2023) developed the MPI (Modeling Path Importance) method, which utilizes node representation learning (node embeddings) in drug-protein interaction networks to prioritize repositioning candidates for AD more effectively. Compared to the standard baseline (BSL) method, MPI identified 20% more substances with evidence of anti-AD action among the top 50 highest-rated drugs (24 drugs for MPI vs. 20 for BSL). Notably, the MPI model detected all four drugs approved by the FDA for the treatment of AD (galantamine, rivastigmine, donepezil, memantine) in this group, while the reference method identified only one. Validation based on MarketScan insurance data confirmed the clinical potential of the selected candidates. It was shown that long-term nicotine use was associated with a significant reduction in the risk of an AD diagnosis (HR = 0.532; *p* < 0.001). Among non-steroidal anti-inflammatory drugs, etodolac showed the strongest protective effect (HR = 0.78; *p* < 0.001), in contrast to flurbiprofen, which did not show statistical significance (HR = 0.95). A harmful effect of trihexyphenidyl, which increased AD risk (HR = 1.71; *p* < 0.001), was also confirmed [13].

Orlenko et al. (2025) developed an explainable machine learning (ML) model, integrating protein–protein interaction data (from the AlzKB database) and drug target information (from the DGIdb database) to detect new epistatic genetic links in Alzheimer’s disease. Using a genomic dataset from the ADSP project, the authors applied a novel Propensity Score Matching method based on PCA to eliminate the influence of population stratification, resulting in a balanced cohort of 22,560 samples. Gene interaction analysis was performed using the BitEpi algorithm, and predictive efficacy was assessed with the XGBoost model. The analysis showed that the baseline model based on known AD genes achieved an ROC AUC score of 63.65%, with variants rs7259620G (PFI = 0.084) and rs769450G (PFI = 0.057) having the greatest impact on prediction. A key discovery was the identification of strong epistatic interactions in the non-coding regions of new candidate genes. Variant pairs within CDC7 showed a high information gain index (alpha = 0.0064), and a model based solely on these new epistatic markers achieved an ROC AUC efficacy of 60.06%, pointing out their potential as new drug targets [14].

A summary of the results of AI-driven drug repositioning in Alzheimer’s disease is presented in Table 1.

### 3.2. Parkinson’s Disease

Parkinson’s disease (PD) is currently the fastest growing neurological disorder in the world in terms of prevalence and social burden, with estimates indicating that the number of patients may double in the coming decades [15]. Despite this, currently available pharmacotherapies focus almost exclusively on alleviating motor symptoms, without offering effective solutions to modify the course of the disease or inhibit the neurodegenerative process [16]. This significant therapeutic gap, combined with the high costs and long development times of de novo drugs, is motivating researchers to use advanced AI methods and RWE data to identify candidates for repositioning that could be brought into clinical practice more quickly [17].

In 2021, Naomi P. Visanji et al. [17] used computer analysis of the available scientific literature to rank drugs according to their potential to inhibit alpha-synuclein (aSyn) oligomerization. Subsequently, to verify these predictions, RWD data were analyzed for correlations between the use of selected drugs and the occurrence of PD. In the in silico stage, using the IBM Watson for Drug Discovery™ (WDD) tool, a group of antihypertensive drugs that could reduce aSyn aggregation was identified. For clinical verification, the authors created a cohort of patients with hypertension using IBM MarketScan™ (IBM Watson Health, Ann Arbor, MI, USA) Research databases. The statistical analysis used univariate and multivariate Cox proportional hazards models, treating drug exposure as a time-dependent variable, with patients using diuretics as the reference group. The models took into account variables such as age at the time of hypertension diagnosis, gender, and comorbidities. The results of multivariate analyses revealed a significant inverse relationship between the time to diagnosis of PD and the use of a combination of angiotensin II receptor blockers (ARBs) and dihydropyridine calcium channel blockers (DHP-CCBs) (HR = 0.55; *p* < 0.01). A protective effect was also observed for combination therapy with angiotensin-converting enzyme inhibitors (ACEi) and diuretics (HR = 0.60; *p* < 0.01). In contrast, an increased risk of disease development was observed with the use of alpha-blockers alone (HR = 1.81; *p* < 0.001) and with the combination of alpha-blockers and calcium channel blockers (HR = 3.17; *p* < 0.05). The obtained results show the potential effectiveness of computational methods in identifying therapies that modify the course of the disease, indicating the potential of combining ARBs with DHP-CCBs in the context of PD.

The study by Daphna Laifenfeld et al. [18] used an emulated clinical trials (ECT)—ECT) framework to identify candidates for drug repositioning in PD. The analysis covered RWD data from two independent US databases: IBM MarketScan (insurance claims data, *n* ≈ 120 million) and IBM Explorys (electronic medical record data, *n* > 60 million). The study included cohorts of patients with late-onset disease (age ≥ 55 years), and the endpoint was a diagnosis of dementia during a two-year follow-up period. The methodology was based on advanced causal inference using inverse probability weighting (IPW) and outcome models to balance confounding factors for 205 tested drugs. The analysis showed that rasagiline was highly effective in reducing the risk of dementia. Compared to a large control group using other anti-Parkinson’s drugs (ATC class N04), rasagiline significantly reduced the incidence of dementia. In the outcome model, a 7 percentage point reduction in the incidence of dementia (effect −0.07) was observed in both the MarketScan database (*p* < 0.001) and the Explorys database (*p* < 0.001). Similar protective effects (7–9% reduction) were observed when compared to a broader group of drugs used in nervous system diseases. In addition, the study identified zolpidem as a drug that significantly reduces the risk of dementia compared to other psycholeptics, showing the potential of the ECT framework in generating and verifying repositioning hypotheses based on RWE data.

In a 2025 study, Maria P. Gorenflo et al. used the Knowledge Graph-Predict system, integrating over 108,000 biomedical entities, which identified amphetamine as the main candidate for repositioning in PD. The accuracy of the model was validated by the presence of 26 drugs already approved for PD among the 30 highest-ranked predictions. RWE verification was performed in a retrospective cohort study based on TriNetX, involving patients over 50 years of age with ADHD. Comparison of the amphetamine-exposed group (*n* = 13,930) with the control group (*n* = 13,848) showed a significant reduction in the risk of PD diagnosis at all time points: the hazard ratio (HR) was 0.59 (95% CI: 0.36–0.98) after 2 years, 0.63 (95% CI: 0.42–0.93) after 4 years, and 0.55 (95% CI: 0.38–0.79) after 6 years. Subgroup analysis revealed a particularly strong protective effect in women (HR at year 4 = 0.24; 95% CI: 0.13–0.45), while in men the differences were not significant. A dose–response relationship was also observed: taking >5 mg of amphetamine was associated with a reduced risk (HR at year 6 = 0.50; 95% CI: 0.25–0.98). Enrichment analysis confirmed the biological basis for these findings, identifying 11 common signaling pathways for amphetamine and PD, including pathways related to dopaminergic synapses and neurodegeneration [19].

A summary of the results of AI-driven drug repositioning in Parkinson’s disease is presented in Table 2.

### 3.3. COVID-19

SARS-CoV-2 spreads primarily through respiratory droplets, entering the respiratory epithelium and occupying lung tissue, which leads to pneumonia and can progress to acute respiratory failure accompanied by multi-organ dysfunction [20]. According to WHO data, the global number of confirmed cases has exceeded 775 million, and the pandemic has generated an unprecedented amount of clinical data. Although a transition to an endemic phase is currently observed—with periodic increases in infections of a milder course resulting from acquired population immunity—the continuous evolution of the virus and the risk to vulnerable groups maintain the necessity of optimizing therapy [21].

An example of a comprehensive approach is a study integrating gene expression profiling (GReX) with clinical data. Voloudakis et al. (2025) utilized a computational drug prioritization model and subjected the selected candidates to verification based on RWE data from the Veterans Health Administration cohort (approx. 9 million patients). Epidemiological analysis demonstrated a statistically significant reduction in COVID-19 incidence among patients taking azathioprine (OR = 0.69; 95% CI: 0.62–0.77) and retinol (OR = 0.81; 95% CI: 0.72–0.92). Interestingly, these results did not overlap with parallel in vitro tests, in which the highest antiviral efficacy was shown by nelfinavir (~95% viral load reduction) and saquinavir (~65% reduction), which were not confirmed in clinical analysis. This discrepancy highlights the necessity of using RWE as a complementary method for preliminary verification algorithmic indications [22].

Conversely, a study by Nam et al. (2023) using a network-based approach addressed the problem of a lack of specific data for a new disease by integrating knowledge of 18 comorbidities and 17 proteins associated with SARS-CoV-2 into a base network (comprising 591 diseases, 26,681 proteins, and 2173 drugs). The applied graph-based semi-supervised learning algorithm selected a list of 30 potential drugs. Their effectiveness was verified in an analysis of RWE data from the Penn Medicine registry. This validation showed that 8 out of 30 (26.7%) candidates selected by AI demonstrated a statistically significant association with the clinical course of COVID-19, supporting the utility of graph methods in conditions of limited information about a new pathogen [23].

A different approach was presented by Rahman et al. (2023), who conducted a so-called virtual clinical trial based on data from the National COVID Cohort Collaborative (N3C) database, which includes over 12 million patients. The authors used advanced causal inference methods, including Propensity Score Weighting and the innovative use of medical code embeddings (SNOMED-CT) using the Node2Vec algorithm. The analysis covered 16 commonly used antidepressants and their impact on the severity of COVID-19 in nearly 242,000 infected patients. The results revealed a complex picture: although the average treatment effect (ATE) for the entire class suggested a slight risk reduction (ATE = −0.076 for the PSW method; *p* < 0.001), the study identified significant heterogeneity—finding patterns indicating that while some antidepressants may be associated with a lower risk of hospitalization, others could potentially increase the risk of complications. This underscores the potential of machine learning-based methods in precisely distinguishing drug effects in large observational datasets [24].

Complementing these reports is a systematic review and meta-analysis conducted by Fico et al. (2022), which synthesized evidence from 29 papers covering computational (in silico), preclinical, and observational (RWE) studies. Quantitative analysis showed that while antidepressants as a whole therapeutic class did not significantly affect mortality risk (RR = 0.94), isolated fluvoxamine demonstrated significant therapeutic potential, being associated with a drastic reduction in the risk of mortality (OR = 0.15; 95% CI: 0.02–0.95). This result contrasted with the group of antipsychotic drugs, whose use was linked to a more than three-fold increase in the risk of severe COVID-19 (RR = 3.66). This study suggests that combining evidence from computational models and clinical data allows for the identification of potential molecules within broader pharmacological groups [25].

A summary of the results of AI-driven drug repositioning in COVID-19 is presented in Table 3.

### 3.4. Type 2 Diabetes

Type 2 diabetes mellitus (T2DM/DM2), which has reached epidemic proportions worldwide, is one of the greatest challenges facing modern medicine, and its multi-organ complications contribute significantly to the increase in global mortality and disability rates [26]. At the same time, it has been observed that the monotherapies currently in use often do not provide sufficient glycemic control or inhibit the development of comorbidities, creating an urgent need to identify new molecules with a more complex profile of action [27].

In a study by Koren et al. (2019), ML techniques (decision trees and neural networks) were used to analyze large medical data sets (Big Data) from the Israeli healthcare system Maccabi Health Services in order to identify drugs that affect glycemic control in type 2 diabetes. The analysis covered 29,540 patients with newly diagnosed diabetes (2005–2016), in whom therapeutic success was defined as achieving an HbA1c level < 6.5% within 90–365 days of starting treatment. Using Propensity Score Matching to eliminate confounding variables, the authors tested 73 drug groups used in comorbidities. The only class of drugs that showed a statistically significant effect on improving glycemic control were alpha-1-adrenergic receptor antagonists (used mainly in benign prostatic hyperplasia). In the group of patients taking these drugs (*n* = 1356), the therapeutic success rate was 61%, compared to 53% in the matched control group (*n* = 1221), which was a significant difference (*p* < 0.0004; test statistic 16.7). An additional analysis in a subgroup of patients treated with biguanides (*n* = 9121) confirmed this beneficial effect (success in patients treated with alpha-1 antagonists: *p* = 0.02). These results suggest the potential for repositioning drugs such as tamsulosin and doxazosin in the treatment of type 2 diabetes [28].

In a retrospective cohort study, Brnabic et al. (2024) used a causal inference framework based on frequentist model averaging (FMA) and ML to assess the impact of dimethyl fumarate (DMF)—an Nrf2 pathway activator used in multiple sclerosis (MS)—on the risk of developing DM2 and its complications. Analyzing data from US insurance claims databases (Merative MarketScan, 2013–2019), a cohort of patients starting DMF therapy (*n* = 3932) was compared with control groups using other disease-modifying drugs: fingolimod (*n* = 1452), glatiramer acetate (*n* = 1989), and teriflunomide (*n* = 935). The results showed a significantly lower risk of developing type 2 diabetes in patients treated with DMF compared to teriflunomide (adjusted hazard ratio, rHR = 0.65; 95% CI: 0.49–0.98). Furthermore, in the same comparison (DMF vs. teriflunomide), a significant reduction in the risk of heart attack (rHR = 0.59; 95% CI: 0.35–0.97) and chronic kidney disease (rHR = 0.52; 95% CI: 0.28–0.86) was observed. Although the differences were not statistically significant when compared to other drugs (fingolimod and glatiramer acetate), this study provides early clinical evidence (RWE) supporting the hypothesis of the potential of Nrf2 pathway activators in the prevention of metabolic diseases [29].

In a study by Gao et al. (2023), an integrated drug-repositioning strategy was proposed to reduce the risk of cataract surgery in patients with diabetes. Using the proprietary KG-Predict system based on knowledge graphs (integrating over 72,000 entities from databases such as DrugBank and GTEx), candidates were selected from among 2650 FDA-approved drugs. This was followed by clinical verification on the TriNetX platform, analyzing EHR data from approximately 800,000 patients with cataracts and diabetes. The results showed that aspirin, melatonin, and ibuprofen were associated with a significant reduction in the risk of cataract surgery in all patient groups (type 1 diabetes, type 2 diabetes, and hyperglycemia) over a 5-, 10-, and 20-year period. For example, in the type 2 diabetes group (*n* = 67,128), aspirin use was associated with a hazard ratio (HR) of 0.72 (95% CI: 0.71–0.75) over a 5-year period. Melatonin showed the strongest long-term protective effect in the hyperglycemia group (*n* = 11,297), achieving an HR of 0.61 (95% CI: 0.55–0.66). Acetylcysteine was effective in type 2 diabetes (HR = 0.65) and hyperglycemia, but not in type 1 diabetes. The mechanism of action of these drugs is likely related to cyclooxygenase-2 (COX-2) inhibition and antioxidant activity [30]. A summary of the results of AI-driven drug repositioning in type 2 diabetes is presented in Table 4.

### 3.5. Neurological Diseases and Mental Disorders

The process of discovering new therapies in neurology and psychiatry is characterized by one of the highest failure rates and exceptionally long product development times [31]. As Mohs and Greig point out, the rejection rate for drugs targeting the central nervous system is significantly higher than in other therapeutic areas, making this sector exceptionally risky for traditional research paths [31]. Gribkoff and Kaczmarek argue that this state of affairs primarily results from the complex and not fully understood pathophysiology of brain diseases and the low translatability of animal models to the human organism, resulting in a lack of clinical efficacy of substances that were promising in the preclinical phase [32]. In the face of these challenges, the strategy of drug repositioning supported by AI algorithms and the analysis of data from real clinical practice represents a key alternative. It allows for the identification of new therapeutic applications based on observable effects in patients, bypassing some limitations of theoretical models. Below are examples of applying this approach in neurodegenerative diseases, emergency states, and addiction disorders.

A pioneering example of integrating phenotypic data with EMRs is the work of Paik et al., in which the ClinDR algorithm, analyzing changes in laboratory results of 530,000 patients, identified terbutaline sulfate as a candidate for treating amyotrophic lateral sclerosis (ALS). Terbutaline (a beta-2 receptor agonist) showed the highest similarity in clinical profile to ursodeoxycholic acid (similarity score = 0.995). In vivo validation in the Danio rerio model confirmed that terbutaline prevented axonal degeneration in a dose-dependent manner (*p* = 2.4 × 10^−13^). Crucially, drug administration after the occurrence of damage (36–48 hpf) resulted in statistically significant “repair” of motor axons (*p* = 2.1 × 10^−11^), and this effect was blocked by beta2-adrenergic antagonists, providing evidence for the potential molecular target of the therapy [33].

In the area of disorders of consciousness, Toker et al. used a deep learning model based on molecular structure, identifying saxagliptin (a DPP-4 inhibitor) as a potential awakening agent. These results were verified in a retrospective study involving 4047 patients in a coma. Analysis showed that patients taking incretin drugs achieved an awakening rate of 76.26%, compared to 64.75% in the non-diabetic control group (difference of 11.5 percentage points; 95% CI for difference: 2.0–21.0%; *p* = 0.0272). Clinically significant, saxagliptin and related drugs showed higher efficacy than amantadine, where the difference in awakening percentage was statistically significant (*p* = 0.0364), and post hoc power was 76% [34].

In psychiatry, the use of AI and RWE allowed for the identification of new therapies in the area of addictions, where effective drugs are lacking. Zhou et al., analyzing EHR data of 72.9 million patients, identified tramadol and olanzapine as drugs significantly increasing the chance of remission in opioid use disorders (OUD). Adjusted Odds Ratio (AOR) for remission was 1.51 for tramadol (95% CI: 1.38–1.66; *p* < 0.001) and 1.90 for olanzapine (95% CI: 1.66–2.18; *p* < 0.001). The study also showed a moderate but significant effect of antidepressants: mirtazapine (AOR = 1.37) and bupropion (AOR = 1.38), which was confirmed by genetic pathway enrichment analysis, indicating common mechanisms (including opioid signaling and serotonin receptor pathways) [35].

In turn, in the case of amphetamine-type stimulant use disorders (ATSUD), Gao et al. used a Knowledge Graphs model and data from the TriNetX network (over 100 million patients), pointing to the potential of ketamine. Cohort analysis showed that ATSUD patients receiving ketamine had a 58% higher probability of remission compared to patients receiving other anesthetics (Hazard Ratio [HR] = 1.58; 95% CI: 1.15–2.17). Moreover, in the subpopulation of patients with co-occurring depression, ketamine showed an advantage over standard antidepressants (HR = 1.51; 95% CI: 1.14–2.01) and over the combination of mirtazapine and bupropion (HR = 1.68; 95% CI: 1.18–2.38) [36].

A summary of the results of studies on using AI based on RWE for drug repositioning in neurology and psychiatry is presented in Table 5.

### 3.6. Autoimmune Diseases

Immune-mediated inflammatory diseases (IMID) are a heterogeneous group of disorders characterized by chronic inflammation and the risk of progressive organ damage. Their etiopathogenesis is multifactorial, with overlapping dysfunctions of the immune system’s signaling pathways playing a key role [37]. The complexity of the pathomechanisms of IMID, including inflammatory bowel diseases (IBDs) and dermatoses such as psoriasis, determines the need for a continuous search for more effective therapeutic solutions [38]. Drug repositioning is therefore a promising alternative, using existing drugs for new therapeutic indications, especially for patients who do not have effective therapies [39].

The study by lawrre et al. (2024) used a machine learning (ML) approach and real-world data (RWD) analysis from Danish medical registries (1996–2019) to identify candidates for drug repurposing to prevent intestinal fibrosis in Crohn’s disease (CD). Analyzing a population of 9179 patients aged ≥65 years (including 1029 cases of surgical intervention), the authors used LASSO regression for optimal selection of predictive features and Marginal Structural Models with IPTW weighting to eliminate errors associated with time-dependent variables. The time-dependent analysis identified 10 drugs that significantly reduced the risk of surgery (taken as a surrogate for severe fibrosis), including fluticasone, fexofenadine, and montelukast. An additional sensitivity analysis confirmed the protective potential of the selected candidates, indicating particularly strong evidence for fluticasone, glucosamine, glycerol triazotate, and clopidogrel as promising therapeutic options for the prevention of CD complications [39].

In a study by Bai et al. (2021), a multi-cohort transcriptomic analysis was performed on 272 colon biopsy samples from 11 public datasets, allowing for the definition of a robust gene signature for ulcerative colitis (UC). By comparing this signature with the expression profiles induced by 781 FDA-approved drugs, atorvastatin was selected as the candidate showing the strongest inverse correlation with disease changes. Clinical verification in a retrospective model, carried out in two independent databases (STARR and Optum Clinformatics DataMart), confirmed a significant association between atorvastatin use and a reduced risk of colectomy. In the STARR cohort (*n* = 827), the hazard ratio (HR) was 0.47 (*p* = 0.03), while in the larger Optum cohort (*n* = 7821), the HR was 0.66 (*p* = 0.03), suggesting the therapeutic potential of this drug in alleviating the severe course of UC [40].

In their study, Patrick et al. (2019) used word embedding on a corpus of over 20 million PubMed abstracts to train a PLS-DA classification model for drug repurposing in skin diseases. The model achieved high average predictive performance (AUROC = 0.93) for 17 immune diseases, identifying budesonide (score = 0.388), hydroxychloroquine (0.388), and leflunomide (0.385) as leading candidates for psoriasis. In silico validation using independent RNA-seq data (92 psoriasis samples, 82 controls) showed a significant enrichment of the molecular targets of these drugs in genes with altered expression (*p* < 1 × 10^−6^). A detailed analysis for budesonide confirmed its impact on key pro-inflammatory cytokines (including CCL2, IL1B), which illustrates the potential of natural language processing (NLP) methods in extracting hidden therapeutic relationships from the literature [41].

A summary of the results of AI-driven drug repositioning in autoimmune diseases is presented in Table 6.

## 4. Discussion

The collected research material suggests that combining computational in silico techniques with the analysis of RWE data represents an emerging framework for the thorough review of the data for new therapeutic indications. The potential of this integrated strategy is reflected in diverse medical areas. However, it is essential to acknowledge that RWE analyses despite using advanced error correction methods remain observational studies, and their findings are sensitive to variable data quality in EHR systems. Analysis of individual studies highlights the close synergy between AI and RWE. In the case of long-term neurodegenerative diseases such as Alzheimer’s Disease (AD) or Parkinson’s Disease (PD), algorithms allowed for the discovery of therapeutic targets that elude classical methods. This is evidenced by the research of Orlenko et al. [14], where machine learning models based on omic data precisely identified new genetic interactions, including those within the CDC7 gene. In PD, the use of knowledge graphs and emulated clinical trials by the teams of Gorenflo et al. [19], Visanji et al. [17], and Laifenfeld et al. [18] enabled the selection of candidates with neuroprotective potential, including rasagiline, amphetamine, or antihypertensive drugs, which was confirmed in the analysis of long-term patient exposure.

The particular utility of the hybrid model (AI supported by RWE) was noted in chronic diseases, such as type 2 diabetes or immune-mediated inflammatory diseases (IMIDs), where it served for the precise assessment of distant endpoints and complications. The works of Shakibfar et al. [39] (Crohn’s disease) and Bai et al. [40] (Ulcerative Colitis) demonstrated that the analysis of medical registries allows for the exploratory verification of the impact of drugs on the risk of surgical interventions, such as intestinal resections. RWE showed similar precision in metabolic studies: the analyses of Koren et al. and Brnabic et al. [29] allowed for the identification of non-obvious therapeutic benefits of drugs used for other indications (e.g., alpha-1 receptor antagonists or dimethyl fumarate), and the study by Gao et al. [30] revealed a protective influence of selected substances on the risk of developing cataracts in diabetics. Furthermore, the application of NLP methods by Patrick et al. [41] in psoriasis proves that semantic literature analysis can effectively indicate candidates with anti-inflammatory effects.

### 4.1. Comparative Analysis of AI Methodologies and Validation Rigor

To address the need for a higher-level synthesis, we categorized the included studies based on their AI approach and the strength of their RWE validation. We identified three primary AI clusters: (1) Network-based and Knowledge Graphs (e.g., MPI, KG-Predict), which prioritize candidates through interaction networks; (2) Transcriptomic-signature matching, focusing on reversing gene expression; and (3) NLP-based literature mining (Table 7).

The strength of the RWE validation varies significantly across the reviewed literature. Studies utilizing causal inference frameworks, such as Emulated Clinical Trials (ECT) or Inverse Probability Weighting (IPW) on large-scale databases (*n* > 1 M), offer the highest degree of preliminary verification. In contrast, studies relying on smaller, localized cohorts or simple descriptive associations represent an emerging but less robust tier of evidence.

### 4.2. Methodological Synthesis

While supervised ML models dominate current RWE validation frameworks due to the necessity of defined clinical endpoints, it is vital to acknowledge the emerging role of advanced, unsupervised methodologies. Our review indicates a ‘supervised bias’ in clinical verification; however, in the broader context of biological systems, unsupervised learning has demonstrated significant efficiency in classification and trend detection. For instance, recent studies have shown that unsupervised approaches, such as t-SNE, can yield superior results across various biochemical systems [42,43,44]. These methods facilitate the identification of high-dimensional patterns without the constraints of predefined labels, offering a powerful tool for the initial stages of drug discovery and patient stratification that could eventually be integrated into more complex RWE pipelines.

### 4.3. Limitations

However, methodological limitations cannot be ignored. It must be remembered that RWE analyses despite using advanced error correction methods such as Propensity Score Matching (PSM) remain observational studies. Their weakness is sensitivity to variable data quality in EHR systems and to confounders not visible in registries, such as diet or lifestyle. For this reason, detected repositioning signals cannot replace randomized clinical trials but instead constitute a solid, data-driven foundation for them. Furthermore, the reliance on a single database (PubMed) for literature identification is a methodological limitation. Although PubMed provides extensive coverage of clinical and biomedical research, this approach may have omitted certain technical or computational advancements published in non-medical databases. In summary, while the interaction of AI with RWE data offers an exploratory approach to data-driven drug discovery, it should be viewed as a preliminary screening layer. Current evidence remains largely retrospective and methodologically heterogeneous, meaning these techniques provide supportive signals rather than a definitive foundation for clinical implementation.

## 5. Conclusions

The conducted literature review suggests that the integration of computational methods (in silico) with RWE data analysis represents an emerging exploratory framework in pharmacology. This combination offers a supportive methodology to address certain limitations of the traditional drug discovery model, with the potential to optimize preliminary research phases. In the process of hypothesis generation, a dominant role is now played by advanced algorithms based on network analysis and NLP, which enable the efficient exploration of non-obvious connections in large-scale biomedical datasets, as confirmed by the identification of new therapeutic indications in areas such as oncology and autoimmune diseases. The conducted literature review indicates that while current validation predominantly relies on supervised AI frameworks, a broader adoption of advanced ML including unsupervised and reinforcement learning is highly beneficial for the next generation of drug-repositioning strategies.

However, it must be emphasized that EHRs have become a key element of this process, moving beyond a purely archival function to become a tool for supporting research hypotheses. Retrospective analyses on large patient cohorts provide a means for the early identification of potential drug efficacy signals. However, due to their observational nature, these findings serve primarily as hypothesis-generating tools to inform, rather than replace, subsequent prospective clinical investigation. Although full utilization of this strategy’s potential requires further standardization of clinical data, our analysis suggests that a tiered approach prioritizing AI-generated candidates validated through large-scale causal inference RWE has the potential to de-risk the preliminary stages of the drug development pipeline.

## Figures and Tables

**Table 1:** Summary of the review of drug-repositioning studies and genetic target identification in Alzheimer’s disease.

Study (Author/Year)	Applied Methodology and Approach	Identified Drugs/ Candidate Genes	Key Validation Results and Conclusions
Taubes et al. (2021) [11]	Precision medicine/Transcriptomic analysis: Focus on the APOE4 genotype; search for a drug reversing pathological gene expression (539-gene signature)	Bumetanide	In vivo (mice): Significant reduction in amyloid plaques (*p* < 0.05), restoration of LTP (*p* < 0.0001), and improvement in cognitive functions. RWE (humans > 65 y.o.): Reduction in AD incidence by 35–75% (statistically significant in two cohorts)
Fang et al. (2022) [12]	Artificial Intelligence (AI)/Network medicine 9: Bayesian algorithm integrating GWAS with multi-omic data	Pioglitazone, Febuxostat, Atenolol	Population: Pioglitazone significantly reduced AD risk vs. control (HR = 0.916) and vs. glipizide (HR = 0.921). In vitro: Pioglitazone reduces GSK3β and CDK5 kinase activity in microglia.
Xiang et al. (2023) [13]	MPI (Modeling Path Importance) method: Use of node representation learning (node embeddings) in interaction networks; comparison with the baseline (BSL) method	Nicotine, Etodolac (Harmful effect of trihexyphenidyl also detected)	Method comparison: MPI identified 20% more anti-AD drugs in the top 50 than BSL (including all 4 FDA-approved drugs). Validation (MarketScan): Nicotine (HR = 0.532) and etodolac (HR = 0.78) significantly reduced the risk of AD diagnosis.
Orlenko et al. (2025) [14]	Explainable ML/Epistatic analysis: XGBoost model, BitEpi algorithm, Propensity Score Matching (PSM), ADSP/AlzKB data	New genetic targets: CDC7, CDC42	Prediction: Baseline model achieved 63.65% ROC AUC Discovery: Strong epistatic interactions identified in non-coding regions of new genes. The model based on CDC7 markers achieved 60.06% ROC AUC

**Table 2:** Summary of the review of research on drug repositioning in PD.

Study (Author/Year)	Applied Methodology and Approach	Identified Drugs	Key Validation Results and Conclusions
Visanji et al. (2021) [17]	Computer analysis of literature (IBM Watson for Drug Discovery) to rank aSyn-inhibiting drugs + RWD verification based on IBM MarketScan (Cox models).	including ARB + DHP-CCB combination, ACEi + diuretics	A significant inverse relationship was demonstrated between the use of ARB and DHP-CCB combinations and PD diagnosis (HR = 0.55; *p* < 0.01) and between ACEi and diuretics (HR = 0.60; *p* < 0.01). Alpha-blockers increased the risk (HR = 1.81).
Laifenfeld et al. (2021) [18]	Framework for emulated clinical trials (ECT) on RWD from IBM MarketScan (*n* ≈ 120 million) and IBM Explorys (*n* > 60 million) databases. Causal inference (IPW, outcome models) was used.	Rasagiline, Zolpidem	Rasagiline significantly reduced the incidence of dementia by 7 percentage points (effect −0.07; *p* < 0.001 in both databases). Zolpidem reduced the risk of dementia compared to other psycholeptics.
Gorenflo et al. (2025) [19]	AI Knowledge Graph-Predict system (108,000 entities) + RWE verification in a retrospective cohort study based on TriNetX (patients with ADHD > 50 years of age).	Amphetamine	Significant reduction in PD risk: HR = 0.59 (2 years), HR = 0.55 (6 years). Strong effect in women (4th year HR = 0.24) and dose dependence (>5 mg: HR = 0.50). Eleven common signaling pathways confirmed.

**Table 3:** Summary of selected studies on the use of AI and RWE in drug repositioning against COVID-19.

Study (Author/Year)	Applied Methodology and Approach	Identified Drugs	Key Validation Results and Conclusions
Voloudakis et al. (2025) [22]	Approach: Translational genomics/GReX. Method: Integration of TWAS (17 tissues) with the LINCS L1000 signature library.	Imiquimod, Nelfinavir, Saquinavir, Everolimus, Azathioprine, Retinol, Nisoldipine.	RWE Validation (VHA ~9 M): Confirmed the action of Azathioprine (OR = 0.69) and Retinol (OR = 0.81). Conclusion: No drug was simultaneously confirmed in both RWE and in vitro models, highlighting the discrepancy between cellular and population models.
Rahman et al. (2023) [24]	Approach: Causal Inference in RWE9. Method: “Virtual clinical trial” using the N3C database (~12 M patients). Use of Propensity Score Weighting and Node2Vec embeddings.	Antidepressants (Analysis of a class of 16 drugs).	RWE Validation: Demonstrated a complex impact of the drug class on hospitalization risk (ATE = −0.076 for the PSW method). Conclusion: Identified drug subgroups with protective effects vs. those increasing risk, emphasizing the need for substance-level rather than class-level analysis.
Nam et al. (2023) [23]	Approach: Network-based complementary linkage. Method: Integration of a “backbone” knowledge network with new COVID-19 data. Graph-based semi-supervised learning (SSL).	8 verified drugs: e.g., Methotrexate, Dexamethasone, Prednisolone, Simvastatin, Acetaminophen, Ibuprofen.	RWE Validation (Penn Medicine ~160 k): 8 out of 30 (26.7%) AI-indicated candidates showed a significant association with clinical outcomes. Conclusion: Successful identification of Dexamethasone validates the utility of network approaches even with incomplete pathogen data.
Fico et al. (2022) [25]	Approach: Systematic Review and Meta-analysis. Method: Synthesis of 29 studies: computational (in silico), preclinical, and clinical (observational/RWE).	Fluvoxamine (positive), Antipsychotics (negative).	Quantitative Results: Fluvoxamine was linked to reduced mortality (OR = 0.15). Antipsychotics were linked to an increased risk of severe course (RR = 3.66). Conclusion: Combining computational and observational data allows for identifying specific effective molecules (like fluvoxamine) despite the lack of a class-wide effect.

**Table 4:** Summary of the review of studies on drug repositioning in DM2.

Study (Author/Year)	Applied Methodology and Approach	Identified Drugs	Key Validation Results and Conclusions
Koren et al. (2019) [28]	ML (decision trees, neural networks) on Big Data (Maccabi Health Services) + propensity score matching (PSM).	Alpha-1 receptor antagonists (e.g., tamsulosin, doxazosin).	Significantly higher therapeutic success rate (HbA1c < 6.5%) in the group treated with alpha-1 antagonists (61%) vs. control (53%) (*p* < 0.0004).
Brnabic et al. (2024) [29]	Causal inference (FMA) and ML on RWD (MarketScan) data. Cohort analysis of patients with MS.	Dimethyl fumarate (DMF)	DMF vs. teriflunomide: lower risk of developing type 2 diabetes (rHR = 0.65), heart attack (rHR = 0.59), and chronic kidney disease (rHR = 0.52).
Gao et al. (2023) [30]	KG-Predict AI system (knowledge graphs) + clinical verification of real-world evidence on the TriNetX platform (approx. 800,000 patients).	Aspirin, melatonin, ibuprofen, acetylcysteine.	Reduction of the risk of cataract surgery in diabetics. Aspirin (T2DM, 5 years): HR = 0.72. Melatonin (hyperglycemia): HR = 0.61. Acetylcysteine effective in T2DM (HR = 0.65) and hyperglycemia.

**Table 5:** Application of AI and RWE methods in drug repositioning in neurology and psychiatry.

Study (Author/Year)	Applied Methodology and Approach	Identified Drugs	Key Validation Results and Conclusions
Paik et al. (2015) [33]	ClinDR: Algorithm integrating genomic data with “clinical signatures” (changes in lab results) from EMRs of 530,000 patients. Analysis of a bipartite graph of drug-disease associations.	Terbutaline sulfate	In vivo validation (Danio rerio model): dose-dependent prevention of axonal degeneration (*p* = 2.4 × 10^−13^). Reversal of damage after symptom onset (*p* = 2.1 × 10^−11^). Conclusion: Terbutaline acts through activation of beta2 receptors, confirmed using an antagonist (butoxamine).
Toker et al. (2025) [34]	Deep Learning: Neural networks analyzing molecular structure (fingerprints). RWE: Retrospective cohort analysis of 4047 patients in a coma (UCLA Health database).	Saxagliptin	Patients treated with incretin drugs had a higher awakening rate (76.26%) than the non-diabetic control group (64.75%; *p* = 0.0272). Significantly higher efficacy compared to standard amantadine treatment (*p* = 0.0364, post hoc power 76%).
Zhou et al. (2021) [35]	DSEG: Predictive system based on a side effect-gene network and protein interactions. RWE: Validation based on IBM Watson Health (72.9 million patients).	Tramadol, Olanzapine, Mirtazapine, Bupropion, Atomoxetine	Olanzapine (schizophrenia treatment) increased the chance of OUD remission: AOR = 1.90 (95% CI 1.66–2.18). Tramadol (analgesic) increased the chance of remission: AOR = 1.51 (95% CI 1.38–1.66). Common mechanisms of action confirmed, including opioid signaling and G-protein activation.
Gao et al. (2024) [36]	KG-Predict: Knowledge Graph model combined with Deep Learning. RWE: Validation in the TriNetX network (>100 million patients) using Target Trial Emulation.	Ketamine	In ATSUD patients, ketamine was associated with more frequent remission compared to other anesthetics (HR = 1.58, 95% CI 1.15–2.17). In the subpopulation with depression, ketamine was more effective than antidepressants (HR = 1.51) and mirtazapine/bupropion (HR = 1.68).

**Table 6:** Summary of the review of research on drug repositioning in immune diseases.

Study (Author/Year)	Applied Methodology and Approach	Identified Drugs	Key Validation Results and Conclusions
Shakibfar et al. (2024) [39]	An approach based on machine learning (ML) and RWD analysis from Danish medical registries (population *n* = 9179). LASSO regression was used for feature selection and structural models (MSM) with IPTW weighting.	including fluticasone, fexofenadine, montelukast, glucosamine, glycerol triazotate, clopidogrel	Time-dependent analysis showed a significant reduction in the risk of surgery (surrogate for fibrosis). Sensitivity analysis confirmed strong protective evidence, particularly for fluticasone and glucosamine.
Bai et al. (2021) [40]	Multicohort transcriptomic analysis (definition of the UC gene signature) combined with clinical verification of RWE in two independent databases (STARR and Optum).	Atorvastatin	A significant association with reduced risk of colectomy was confirmed. In the STARR cohort (*n* = 827), the HR was 0.47 (*p* = 0.03), and in the Optum cohort (*n* = 7821), the HR was 0.66 (*p* = 0.03).
Patrick et al. (2019) [41]	NLP technique (word embedding) on >20 million PubMed abstracts for training the PLS-DA classification model. In silico validation on independent RNA-seq data.	including budesonide, hydroxychloroquine, leflunomide (for psoriasis)	The model achieved high predictive accuracy (AUROC = 0.93). Molecular drug targets showed significant enrichment in genes with altered expression (*p* < 1 × 10^−6^), validating the potential of NLP methods.

**Table 7:** Analytical classification and evidence strength of selected repositioning studies.

Evidence Tier	AI Methodology	RWE Validation Quality	Key Examples
Tier 1 (High)	Knowledge Graphs/Deep Learning	Causal inference (ECT, IPW, TTE), *n* > 1 M, multi-database validation	Laifenfeld et al. [18], Rahman et al. [24]
Tier 2 (Moderate)	Network analysis/Transcriptomics	Matched cohort studies (PSM/IPTW), *n* > 10 k	Taubes et al. [11], Koren et al. [28]
Tier 3 (Emerging)	NLP/Semantic Mining	Association studies, localized EMRs, *n* < 10 k	Patrick et al. [41], Bai et al. [40]

## Data Availability

The original contributions presented in this study are included in the article. Further inquiries can be directed to the corresponding author.

## Abbreviations

The following abbreviations are used in this manuscript:

ACEi	Angiotensin-Converting Enzyme inhibitors
AD	Alzheimer’s Disease
ADHD	Attention-Deficit/Hyperactivity Disorder
AI	Artificial Intelligence
ALS	Amyotrophic Lateral Sclerosis
AOR	Adjusted Odds Ratio
ARB	Angiotensin II Receptor Blockers
ARG	Alzheimer’s Risk Genes
ATE	Average Treatment Effect
ATSUD	Amphetamine-Type Stimulant Use Disorder
AUC	Area Under the curve
aSyn	Alpha-synuclein
BSL	Baseline
CD	Crohn’s Disease
CI	Confidence Interval
COX-2	Cyclooxygenase-2
DHP-CCB	Dihydropyridine Calcium Channel Blocker
DMF	Dimethyl Fumarate
DPP-4	Dipeptidyl Peptidase-4
ECT	Emulated Clinical Trials
EHR/EMR	Electronic Health Record/Electronic Medical Record
FDA	Food and Drug Administration
FMA	Frequentist Model Averaging
GReX	Gene Expression Profiling
GWAS	Genome-Wide Association Study
HbA1c	Glycated hemoglobin
HR	Hazard Ratio
IBD	Inflammatory Bowel Diseases
IMID	Immune-Mediated Inflammatory Diseases
IPTW	Inverse Probability of Treatment Weighting
IPW	Inverse Probability Weighting
LASSO	Least Absolute Shrinkage and Selection Operator
LTP	Long-Term Potentiation
MeSH	Medical Subject Headings
ML	Machine Learning
MPI	Modeling Path Importance
MS	Multiple Sclerosis
MSM	Marginal Structural Models
NLP	Natural Language Processing
OR	Odds Ratio
OUD	Opioid Use Disorders
PCA	Principal Component Analysis
PD	1. Pharmacodynamic; 2. Parkinson’s Disease
PK	Pharmacokinetic
PSM	Propensity Score Matching
PSW	Propensity Score Weighting
RR	Relative Risk
RWE/RWD	Real-World Evidence/Real-World Data
SSL	Semi-Supervised Learning
T2DM	Type 2 Diabetes Mellitus
UC	Ulcerative Colitis
WHO	World Health Organization
